# Segregation of the Brain into Gray and White Matter: A Design Minimizing Conduction Delays

**DOI:** 10.1371/journal.pcbi.0010078

**Published:** 2005-12-30

**Authors:** Quan Wen, Dmitri B Chklovskii

**Affiliations:** 1 Department of Physics and Astronomy, State University of New York at Stony Brook, Stony Brook, New York, United States of America; 2 Cold Spring Harbor Laboratory, Cold Spring Harbor, New York, United States of America; University College London, United Kingdom

## Abstract

A ubiquitous feature of the vertebrate anatomy is the segregation of the brain into white and gray matter. Assuming that evolution maximized brain functionality, what is the reason for such segregation? To answer this question, we posit that brain functionality requires high interconnectivity and short conduction delays. Based on this assumption we searched for the optimal brain architecture by comparing different candidate designs. We found that the optimal design depends on the number of neurons, interneuronal connectivity, and axon diameter. In particular, the requirement to connect neurons with many fast axons drives the segregation of the brain into white and gray matter. These results provide a possible explanation for the structure of various regions of the vertebrate brain, such as the mammalian neocortex and neostriatum, the avian telencephalon, and the spinal cord.

## Synopsis

Vertebrate brains generally contain two kinds of tissue: gray matter and white matter. Gray matter contains local networks of neurons that are wired by dendrites and mostly nonmyelinated local axons. White matter contains long-range axons that implement global communication via often myelinated axons. What is the evolutionary advantage of segregating the brain into white and gray matter rather than intermixing them? In this study, the authors postulate that brain functionality benefits from high synaptic connectivity and short conduction delays—the time required for a signal from one neuron soma to reach another. Using this postulate, they show quantitatively that the existence of many fast, long-range axons drives the segregation of the brain into gray and white matter. The theory not only provides a possible explanation for the structure of various brain regions such as cerebral cortex, neostriatum, and spinal cord, but also makes several testable predictions such as the scaling estimate of the cortical thickness.

## Introduction

A ubiquitous feature of the vertebrate brain is its segregation into white and gray matter (http://www.brainmuseum.org). Gray matter contains neuron somata, synapses, and local wiring, such as dendrites and mostly nonmyelinated axons. White matter contains global, and in large brains mostly myelinated, axons that implement global communication. What is the evolutionary advantage of such segregation [1]? Networks with the same local and global connectivity could be wired so that global and local connections are finely intermixed. Since such design is not observed, and invoking an evolutionary accident as an explanation has agnostic flavor, we searched for an explanation based on the optimization approach [2–6], which is rooted in the evolutionary theory [7–9].

We started with the assumption that evolution “tinkered” with brain design to maximize its functionality. Brain functionality must benefit from higher synaptic connectivity, because synaptic connections are central for information processing as well as learning and memory, thought to manifest in synaptic modifications [10,11]. However, increasing connectivity requires adding wiring to the network, which comes at a cost. The cost of wiring is due to metabolic energy required for maintenance and conduction [12–15], guidance mechanisms in development [16], conduction time delays and attenuation [17,18], and wiring volume [6].

Two pioneering studies, by Ruppin et al. [19] and Murre and Sturdy [20], have proposed that the segregation of white and gray matter could be a consequence of minimizing the wiring volume. They modeled the brain by a network consisting of local and global connections, which give rise to gray and white matter correspondingly. Although wiring volume minimization is an important factor in the evolution of brain design, their results remain inconclusive because predictions of the volume minimization model for the present problem are not robust and are difficult to compare with empirical observations (see Discussion).

In this paper, we adopted the model of connectivity introduced in Ruppin et al. [19] and Murre and Sturdy [20], including local and global connections, but minimized the conduction delay, i.e., the time that takes a signal (such as action potential and/or graded potential) to travel from one neuron's soma to another. To see that high connectivity and short conduction delay are competing requirements, note that adding wiring to the network increases not only its volume, but also the distance between neurons. In turn, this requires longer wiring, which, for the same conduction velocity, introduces longer delays. Longer delays are detrimental because fewer computational steps can be performed within the time frame imposed on animals by the environment, making the brain a less powerful computational machine [12].

We show that the competing requirements for high connectivity and short conduction delay may lead naturally to the observed architecture of vertebrate brain as seen in mammalian neocortex and bird telencephalon. As in any other theoretical analysis, we make several major assumptions. First, given that exact connectivity is not known, we characterized the interneuronal connectivity statistically by requiring a fixed number of connections per neuron. Second, although conduction delays are known to differ between connections, we minimized the mean conduction delay. Finally, it is likely that, in the course of evolution, minimization of conduction delay was accompanied by the increase in connectivity. However, it is not known how to quantify the benefits of increased connectivity in comparison with conduction delay increase. Therefore, we adopted a mathematically sound approach of minimizing conduction delay while keeping network connectivity fixed.

To obtain quantitative results, we used two analytical (nonnumerical) tools borrowed from theoretical physics. First, most of the derivations were done using the scaling approach. In this approach, a relationship between variables takes the form of proportionality rather than equality. In other words, numerical factors of order one are ignored. One can manipulate and combine such proportionality relationships and still get an estimate that is correct by an order of magnitude. A long history of successful applications of the scaling approach supports its validity. Second, we used a perturbation theory approach, which is helpful when the exact analytical solution to a problem is unavailable. In this approach, a simpler problem is solved exactly. Then the exact solution is modified to fit the actual problem by taking advantage of the fact that such modification is minor. Again, the long history of this approach supports its validity, as long as the difference between the exactly solvable and the actual problem is characterized by a parameter that is much smaller than one.

We present our theory in Results, which is organized into seven sections. In the first, we consider competing requirements between small conduction delays and high connectivity in local circuits. We show that local conduction delay limits the size of the local network with all-to-all potential connectivity to the size of the cortical column. The second section models full brain architecture as a small-world network, which combines high local connectivity with small conduction delay. We derive a simple estimate of conduction delay in global connections as a function of the number of neurons. In the third section, we consider spatially integrating local and global connections. We argue that mixing local and global connections substantially increases local conduction delay, while the global conduction delay may be unaffected. In the fourth section, by minimizing local conduction delay we derive a condition under which white/gray matter segregation reduces conduction time delays. The fifth section gives a necessary condition for the segregated design to be optimal, and an example of such design is given in the sixth section. Finally, the seventh section restates our results in terms of the numbers of neurons, interneuronal connectivity, and axon diameter.

## Results

### Conduction Delays Limit the Size of a Highly Connected Network

We begin by considering the time delay in the local circuits of neocortex, because their mode of operation—thought to involve recurrent computations [21,22]—seems most sensitive to the detrimental impact of time delay. We derive a scaling relationship between local conduction delay and the number of neurons that can have all-to-all potential connectivity. By assuming that the tolerable delay is on the order of a millisecond, we show that the maximum size of such network is close to that of the cortical column.

Local cortical circuits may be viewed as a network of *n* neurons with all-to-all potential synaptic connectivity, meaning that the axons and dendrites of most neurons come close enough to form a synapse [23–25]. In the following we do not distinguish between axons and dendrites in local circuits, and we refer to them as “local wires.” Mathematical symbols used in this paper are shown in Table 1. The mean conduction delay *t* in local circuits is given by the average path length between two connected neurons (via potential synapses), ℓ*,* divided by the conduction velocity, *s*:

**Table 1:**
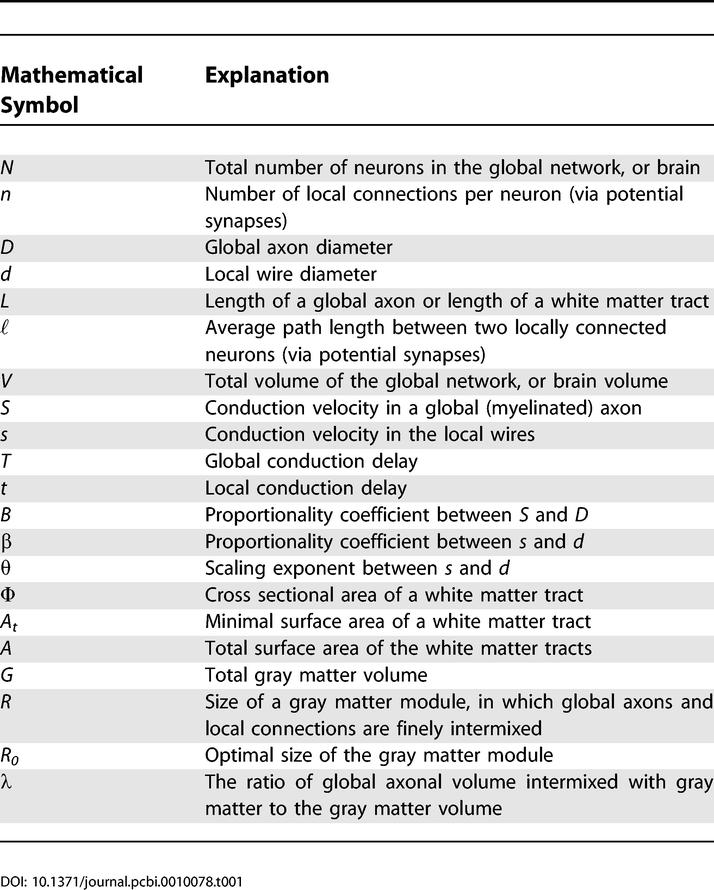
Mathematical Symbols Used in the Main Text


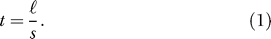


Experimental measurements [26,27] and theoretical arguments [28,29] suggest that conduction velocity, *s,* scales sublinearly with the diameter, *d,* of local wires (nonmyelinated axons and dendrites):


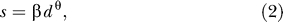


where *β* is a constant coefficient and *θ* is a positive power smaller than one (however, see [30]). By combining Equations 1 and 2, we arrive at the expression for the conduction delay:


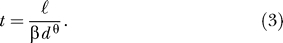


Equation 3 may give an impression that the conduction delay decreases monotonically with wire diameter *d*. But this is not necessarily the case because ℓ can be a function of *d*. The following argument [17] shows that the conduction delay, *t,* as a function of wire diameter, *d,* has a minimum (provided 0 < *θ* < 1), which defines the optimal wire diameter. Given the branching structure of axons and dendrites and uniform distribution of neurons, ℓ can be approximated by the linear size of the network [6], which can be easily estimated in the two limiting cases. In the limit when the wire diameter approaches zero, all the nonwire components (such as synapses) are compressed together and take up the space vacated by shrinking wires. Because the volume of the network approaches the volume of the nonwire components, which is constant, the conduction delay diverges as 1/*d^ θ^* according to Equation 3 [17].

In the opposite limit when the wire diameter is large, the network volume is determined mostly by the wiring [17]. Because wires run in all directions, they must get longer as they get thicker, and the linear size of the network grows proportionally to the wire diameter. Then, according to Equation 3, the conduction delay increases as *d* ^1-*θ*^. Therefore, conduction delay is minimized by the optimal wire diameter, for which the nonwire occupies a fixed fraction of the neuropil volume [17] (see also the first section in Materials and Methods). As a result, the optimal volume of the network is of the same order as the nonwire volume. Assuming that nonwire consists mostly of synaptic components, such as axonal boutons and spine heads, the optimal network volume is of the same order as the total synaptic volume. Therefore, the local network volume is given by:


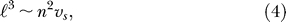


where *v_s_* is the average synapse volume and *n* is the total number of neurons in the local network. (In a network with all-to-all connectivity, *n* is also the number of local connections made by a neuron via potential synapses.) For the sake of clarity, we ignore the fact that only a fraction (0.1–0.3) of potential synapses are converted into actual synapses [23]. Such numerical factors are ignored in the equations of the main text of this paper, but can be included straightforwardly (see the first section in Materials and Methods). One consequence of Equation 4 is that the optimal wire diameter is on the same order of magnitude as the synaptic linear size, consistent with anatomical observations [31]:


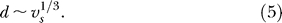


By using Equations 3–5 and assuming *θ* = 1/2, suggested by the cable theory [28,29], we find that the smallest possible mean conduction delay in local networks is given by


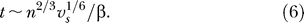


As the smallest possible conduction delay grows with the number of neurons in the network, fixing conduction delay imposes a constraint on the maximum size of the network. It seems reasonable to assume that the biggest tolerable conduction delay is on the order of a millisecond, a time scale corresponding to physiological events such as the extent of an action potential and the rise-time of an excitatory postsynaptic potential [32]. This time scale could be dictated by the metabolic costs [33]. If we approximate the synaptic volume at a fraction of a cubic micrometer, and *β* ~ 1 m/s μm^−1/2^ [28,34,35], the maximum number of neurons in the all-to-all connected network is on the order of 10^4^. This corresponds to roughly the size of a cortical column, which is then the largest network that can combine all-to-all potential synaptic connectivity with tolerable conduction delay.

### Small-World Network Combines High Local Connectivity with Small Conduction Delay

Human neocortex contains about 10^10^ neurons—many more than could possibly be wired in an all-to-all fashion with a physiologically tolerable conduction delay. In particular, substituting this neuron number into Equation 6, we find that the delay would be on the order of seconds—clearly too slow. Given that the brain is too large to combine high interconnectivity with short conduction delay [36,37] how can it maintain high functionality? In this section, we consider the architecture of the brain as a whole and show that much shorter global conduction delay can be achieved by sacrificing all-to-all connectivity.

Anatomical evidence suggests that the brain maintains short conduction delays by implementing sparse global interconnectivity while preserving high local interconnectivity [31]. Such design resembles the small-world network [38], as has been noticed by several authors [39–42]. In a small-world network, a high degree of clustering (the probability of a connection between two neighbors of one neuron) is combined with a small network diameter (the average number of synapses on the shortest path connecting any two neurons). In a neurobiological context this means a combination of high computational power in local circuits with fast global communication [31,36,37,39,40,42]. Thus it is not surprising that evolution adopted this architecture when the size of the network made all-to-all connectivity impractical [36,39,43–45].

How fast could global connections be? Global conduction delay *T* in a connection of length *L* with conduction velocity *S* is given by


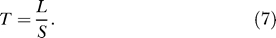


Here and below, upper-case letters are reserved for parameters of global connections and lower-case letters for parameters of local connections. In big brains, global axons are mostly myelinated as would be expected, given higher demand on their conduction velocity (unpublished data) [28]. In myelinated axons, conduction velocity *S* scales linearly with diameter [28,46], *D* as


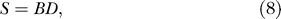


where *B* is a proportionality coefficient. Combining Equations 7 and 8, we find that the conduction delay is given by


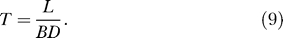


The average length of global connections is given by


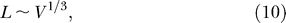


where *V* is brain volume. In turn, brain volume can be estimated by adopting the following model. Based on anatomical data [31], we assume that most neurons send one global connection to another local network in the brain. Initially, we ignore the volume occupied by local connections. We denote the number of neurons in the brain as *N,* which can be much larger than the number of local connections (via potential synapses) per neuron, *n*. Global connections have length *L* and diameter *D*. Thus the total volume of the brain can be approximated as


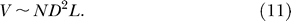


Combining Equations 10 and 11, we find


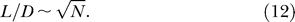


Substituting this expression into Equation 9, we obtain


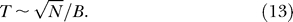


Equation 13 can be used to estimate conduction delay in global axons. By substituting *B* ~ 5 m/s μm^−1^ [46,47] and the number of neurons in human neocortex, *N* ~ 10^10^, we find that the delay is around 20 ms. Compared with the several-second delay expected in a human brain if it had all-to-all connectivity, this is a significant improvement. For the mouse neocortex, by substituting *N* ~ 10^7^ we find that the delay is around 0.6 ms. This is much better than the 50-ms delay expected, according to Equation 6, if the mouse cortex had all-to-all connectivity. As these estimates are based on the scaling approach, they are reliable only up to an order of magnitude. Yet, they demonstrate that sparse global connections can be much faster than a fully connected network with a comparable number of neurons.

### Combining Local and Global Connections Increases Conduction Delays

After having considered conduction delays in local and global connections separately, now we are in a position to analyze how they are combined in the brain. Here we argue that the main difficulty in integration arises when introducing global connections into local networks.

We adopt a model combining both local and global connections proposed by Ruppin et al. [19] and Murre and Sturdy [20]. In this model, each neuron connects (via potential synapses in our case) with *n* local neurons and sends a global axon to another arbitrarily chosen local network in the brain. For simplicity, we neglect specificity and assume that local connections are made with nearest *n* neurons located in a sphere of radius ℓ centered on a given neuron, where ℓ is given by Equation 4. Although local and global connections may be highly specific [22,48–50], this approximation is sufficient to understand brain segregation into white and gray matter.

The effect of combining local and global connections on the conduction delays can be analyzed in two steps. First, consider the effect of introducing local connections into the network of global connections. This leads to the swelling of the brain volume beyond that in Equation 11. Thus, global axons must be longer, and Equation 13 gives only the lower bound for global conduction delay (see the second section in Materials and Methods). Yet the increase in the global conduction delay caused by the swelling of network can be offset via speeding up global axons by making them thicker, (Equation 8). We show in the second section in Materials and Methods that the global network can absorb local connections and preserve the required global conduction delay.

Second, introduction of global connections into local circuits increases local conduction delay and is impossible to compensate by making local connections thicker (see the third section in Materials and Methods). While conduction velocity depends linearly on the global myelinated axon diameter (Equation 8), it scales sublinearly with the local wire diameter (Equation 2). Thus, the smallest possible mean local conduction delay increases when more global connections are mixed with local connections. To describe this quantitatively, we introduce the ratio of global axon volume that is finely intermixed with local connections to the initial unperturbed gray matter (i.e., total local circuits) volume, *λ*. When *λ* is much smaller than one, we can argue that the initial minimum local conduction delay is only slightly affected by the penetration of global connections in the gray matter. As shown in the third section in Materials and Methods, because of intermixing global connections and local connections, the increase in local conduction delay, Δ*t,* is proportional to the ratio *λ*:


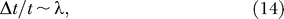


where *t* is conduction delay in unperturbed local circuits given by Equation 6. As before, numerical factors are neglected in the spirit of the scaling estimate*.*

According to our original assumption, brain functionality is maximized when conduction delay is minimized. According to Equation 14, the smallest possible conduction delay in local circuits is achieved when *λ =* 0, i.e., when global and local connections are fully segregated. But full segregation does not lead to a feasible design, because global connections originate and terminate on neurons in local circuits. Thus, we must find a design that spatially integrates local and global connections.

We note that minimization of local and global conduction delays are competing desiderata, as can be illustrated by varying the global axon diameter, *D*. Increasing *D* speeds up signal propagation along global connections and, therefore, reduces global conduction delay. Yet, thicker global axons are detrimental for local conduction delay because of an increase in *λ* (Equation 14). As the relative contributions to functionality of conduction delays in local and global connections are unknown, we searched for the optimal design that minimizes local conduction delay as a function of *D*. Our analysis begins with considering small values of *D*, i.e., *λ* ≪ 1.

### Comparison of the Homogeneous Design and Designs with Gray and White Matter Segregation

In order to determine the optimal design we need to compare local conduction delays in different designs combining gray and white matter. In general, this problem is difficult to solve analytically. Yet, when global connections that are intermixed with the gray matter take less volume than does local, i.e., *λ* ≪ 1, the perturbation theory approach allows us to compare local conduction delays in homogeneous design (HD), in which gray matter and white matter are finely intermixed, to designs in which gray and white matter are segregated.

**Figure 1:**
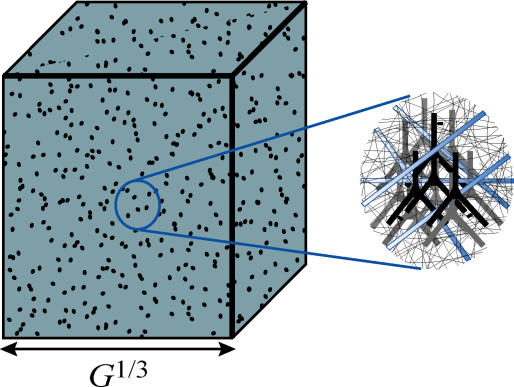
Homogeneous Design In HD, local and global connections are uniformly and finely intermixed. Inset shows a typical local network containing local axons (thin gray lines) and dendrites (gray and black tree-like structures), and global axons (thick, light-blue lines spanning the whole circle) that perforate gray matter. When the volume of global axons is small, the linear size of the network can be approximated as *G* ^1/3^.


In HD, local and global connections are finely and uniformly intermixed (Figure 1). Then, according to Equation 14, the relative conduction delay increase due to the penetration of global axons of diameter *D* in the gray matter is given by

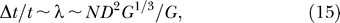


where *N* is the total number of neurons in the network. In this expression, we use Equation 11 for the volume of global connections and the fact that the average length of global axons is given by the linear size of the network, which, for a small *λ,* is given by the linear size of gray matter, *G* ^1/3^. We note that the perturbation approach remains valid while the relative conduction increase in HD is less than one, i.e., *ND* ^2^ ≪ *G* ^2/3^.

**Figure 2:**
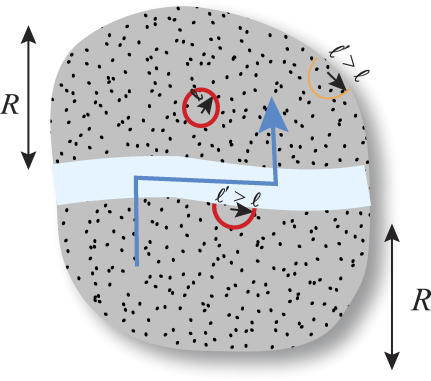
Boundary Effects in the Gray Matter The red full circle illustrates the local connection sphere of a neuron that does not experience the boundary effect. Neurons near external boundary must inflate their local connection sphere to implement the required local connectivity, as illustrated by thin yellow semicircle. Neurons near white matter tracts penetrating gray matter must also inflate their local connection sphere to implement the required local connectivity, as illustrated by the thick red semicircle. Blue line with arrowhead shows typical routing of global axons. *R* is the size of gray matter modules, where global and local connections are finely intermixed.

Another contribution to the mean local conduction delay comes from the boundary effect. Recall that the model requires each neuron in the gray matter to establish connections with *n* nearest neighbors. If a neuron is far from the boundary of the gray matter, these connections can be implemented in a sphere of radius ℓ given by Equation 4 (Figure 2). Yet neurons within distance ℓ of the gray matter boundary cannot find *n* neighbors within the sphere of the same size. Therefore, the radius of the local connections sphere must be expanded to find *n* nearest neighbors (Figure 2).

Expanding the range of local connections for neurons near the boundary increases average local conduction delay. The fraction of neurons that experience the boundary effect is proportional to the volume within distance ℓ from the boundary. As the boundary area in HD is given by *G* ^2/3^, the fraction of affected neurons is given by ℓ*G* ^2/3^/*G* ~ ℓ/*G* ^1/3^, which is less than one because the linear size of the gray matter *G* ^1/3^ ≫ ℓ. Since the relative increase in delay for each neuron in the affected volume is of order one, this expression also gives a relative increase in the average local conduction delay. As this boundary effect is determined by the external boundary, it is independent of the design and can be ignored. Yet, the logic of this calculation will be used in the following to estimate the effect of gray and white matter boundary on local conduction delay.

Can segregation of gray and white matter reduce local conduction delay in HD? In HD, global axons are straight and are finely intermixed with the local connections. The contribution of global axons to local conduction delays could be reduced by decreasing the length of global axonal segments within the gray matter, according to Equation 14. Rather than connecting neurons with a straight axon, a typical global axon would go toward the nearest white matter tract (region occupied only by global axons) and travel in the white matter until it is close to the target neuron. Then the axon would leave the white matter and traverse the gray matter toward its target (Figure 2). Such routing may increase the length of global axons, but it would minimize impact on local conduction delays.

To calculate the relative local delay increase in the segregated design we estimate the relative volume of global axons in the gray matter, *λ*. We introduce the mean distance between a neuron and the nearest white matter tract, *R,* which also gives the linear size of gray matter modules (Figure 2). Then the relative volume of nonfasciculated global axons inside the gray matter in the segregated design is given by


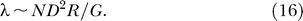


Comparing Equation 16 with Equation 15, one can see that segregation may be advantageous to HD if *R* ≪ *G* ^1/3^. In other words, introducing a sufficient number of white matter tracts into the gray matter may reduce the length of nonfasciculated global axonal segments in the gray matter and, hence, the local conduction delay.

Although segregation of gray and white matter may reduce local conduction delay, it has a disadvantage compared to HD in that it may induce a larger boundary effect because of the white matter tracts inside the gray matter. This effect is similar to the external boundary effect in HD, but it cannot be ignored, because it is different for different designs. If a neuron is far from the gray and white matter interface, its local connections can be implemented in the sphere of radius ℓ (Equation 4; Figure 2). If a neuron is close to the interface, the white matter occupies part of the sphere, meaning that the local sphere radius ℓ must be expanded so that a neuron can still find its *n* nearest neighbors (Figure 2). Therefore, whether the segregated design is preferred or not depends on whether the relative local conduction delay increase through the boundary effect is much smaller than the local delay increase in HD (Equation 15).

To evaluate the mean local conduction delay increase through the boundary effect in the segregated design, we need to specify the geometry of the white matter tracts, because the boundary effect generally depends on the surface area of the tracts. For a typical tract that spans the whole brain (i.e., has length *L*), we can relate its minimal surface area *A_t_* to its cross-sectional area, Φ:


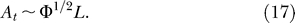


In turn, the cross-sectional area of a tract depends on the global axon diameter *D,* and one may conjecture that whether the segregated designs are advantageous or not depends on *D*. Indeed, we can formulate the following theorem, which is valid to the first order of *ND* ^2^/*G* ^2/3^ (Equation 15) and while our perturbation approach is valid (i.e., provided *ND* ^2^ ≪ *G*/ℓ, as will be shown later).

#### Theorem 1.

In the regime ND^2^ ≪ ℓ^2^, local conduction delays in the optimal segregated design and HD are equivalent. In the regime ND^2^ ≫ ℓ^2^, there is at least one segregated design with local delays less than those in HD.

**Figure 3:**
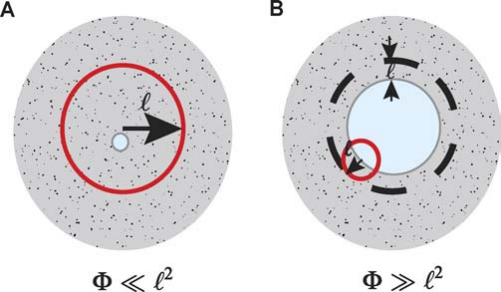
Boundary Effect Induced by White Matter Tracts with Different Cross-Sectional Areas (A) In the case Φ ≪ ℓ^2^, two dimensions of the white matter tracts (shown in white) can be much smaller than ℓ. Red circle illustrates local connection sphere of a neuron. (B) In the case Φ ≫ ℓ^2^, neurons within distance ℓ from the white matter tract experience the boundary effect.

To prove the first part of the theorem, we calculate the local conduction delay through the boundary effect in the segregated designs and compare it with HD. The length of the global tract segment inside the local sphere is ℓ. The other two dimensions of global tracts are much less than ℓ (Figure 3A), as the minimal boundary effect is achieved by the minimal surface area in Equation 17. Since the total cross-sectional area of the global tracts is *ND* ^2^ ≪ ℓ^2^, each tract's cross-sectional area, Φ*_i_,* is much less than the cross-sectional area of the local connection sphere (Figure 3A). Inclusion of such a tract into a local sphere increases its radius to (ℓ^2^ + Φ*_i_*)^1/2^. Then, the relative increase in the local conduction delay for neurons in that sphere is [(ℓ^2^ + Φ*_i_*)^1/2^ − ℓ]/ℓ ≃ Φ*_i_* /ℓ^2^ ≪ 1.

Now we add up conduction delays contributed by all the tracts to neurons in affected spheres. As the number of spheres affected by one tract is given by *L*/ℓ*,* the fraction of neurons experiencing the boundary effect induced by one tract is given by ℓ^2^ *L*/*G,* and the relative local conduction delay increase is given by (Φ*_i_*/ℓ^2^)ℓ^2^ *L*/*G* ~ Φ*_i_*/*G* ^2/3^. The total relative increase in local delay is the sum of the boundary effects induced by different tracts,


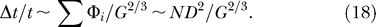


Notice that even if there are multiple tracts within the local connection sphere (i.e., the sphere radius can be larger than ℓ), the above result is still correct.

By comparing local conduction delay increase for segregated designs (Equation 18) with that for HD (Equation 15), one can see that they are the same. Therefore, when *ND* ^2^ ≪ ℓ^2^, the optimal segregated designs and HD are equivalent to the first order of *ND* ^2^/*G* ^2/3^.

To prove the second part of the theorem (the *ND* ^2^ ≫ ℓ^2^ regime), we specify a segregated design with smaller local delays than that in HD. In such a design, global axons belong to *M* (*M* ≫ 1) tracts with cross-sectional area Φ ≫ ℓ^2^ each and length *L* ~ *G* ^1/3^. The distance between two tracts is much larger than ℓ. Then, the total affected neuropil volume through the boundary effect is the product of the total surface area of the tracts, *M*Φ^1/2^ *G* ^1/3^, and ℓ. For a typical neuron within the affected volume, a fraction of its local connection sphere with volume ~ℓ^3^ is occupied by the white matter tract, as illustrated in Figure 3B. To implement the required local connectivity, the local sphere radius ℓ should expand by a numerical factor of order one.

Next, we add up the relative local delay increase induced by all global tracts affecting all the neurons in a volume, given by ℓ*M*Φ^1/2^ *G* ^1/3^/*G*. Because the total cross-sectional area *M*Φ ~ *ND* ^2^, the relative local delay increase is


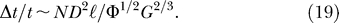


By comparing relative conduction delay in segregated design (Equation 19) with that in HD (Equation 15), one can see that because Φ ≫ ℓ^2^ as specified, segregated design is advantageous in the regime *ND* ^2^ ≪ *G* ^2/3^.

Although in the regime *ND* ^2^ ≫ *G* ^2/3^ we do not have a closed-form expression for the local conduction delay in HD, we can still show that it has longer conduction delays than the segregated design. We show in the third section in Materials and Methods that local conduction delay in HD is a monotonically increasing function of *λ,* and hence is a monotonically increasing function of *ND* ^2^. Thus, the relative delay increase in HD exceeds one when *ND* ^2^ ≫ *G* ^2/3^. Yet, in the regime *ND* ^2^ ≫ *G* ^2/3^, the relative local delay increase in a segregated design can still be much smaller than one. To prove this, we note that in a segregated design, the local conduction delay increase because of the nonfasciculated global axons intermixed with gray matter, i.e., *λ* ~ *ND* ^2^ *R*/*G* (Equation 16), can be much smaller than one, if *R* ≪ *G* ^1/3^.

In addition, the relative local delay increase through the boundary effect can also be much smaller than one. To see this, we specify the tracts in such a way that the total surface area of the white matter tracts is the surface area of the gray matter *G*/*R*. Then, using an analysis similar to that illustrated in Figure 3B, the relative local delay increase through the boundary effect is given by ℓ*G*/(*RG*), which can be much smaller than one if ℓ/*R* ≪ 1. We note that λ ≪ 1 and *R* ≫ ℓ could both be satisfied if *ND* ^2^ ≪ *G*/ℓ. Thus, when *ND* ^2^ ≫ *G* ^2/3^ and *ND* ^2^ ≪ *G*/ℓ, there is at least one segregated design with a local delay less than that in HD.

Having considered both the *ND* ^2^ ≪ *G* ^2/3^ regime and *ND* ^2^ ≫ *G* ^2/3^ regime, we have proven the second part the theorem.

### Optimality Condition for Segregated Designs

In the previous section, we showed that in the regime *ND* ^2^ ≫ ℓ^2^, there is at least one segregated design with local conduction delay shorter than that in HD. However, we did not specify which design is the optimal one. In this section, we give a necessary condition for a segregated design to be optimal in the regime *ND* ^2^ ≫ ℓ^2^ and *ND* ^2^ ≪ *G*/ℓ.

As the advantage of segregation becomes apparent when the total cross-section of global axons *ND* ^2^ ~ ℓ^2^, it is natural to expect that a similar condition defines the optimal gray matter module size *R*_0_, which minimizes local conduction delays. In other words, the number of neurons in the gray matter module is such that the total cross-sectional area of their global axons is given by ℓ^2^. As the number of neurons in the sphere of radius *R*_0_ is ℓ^2^/*D* ^2^ and the number of neurons in the sphere of radius ℓ is *n*, we have


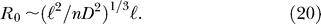


Thus, we can formulate the following theorem:

#### Theorem 2.

In the regime ND^2^ ≫ ℓ^2^ and ND^2^ ≪ G/ℓ, the minimum local conduction delay is achieved by the segregated design with the gray matter module containing ℓ^2^/D^2^ neurons.

To prove this theorem, we consider designs with gray matter module size smaller and greater than *R*_0_, and show that they have a local conduction delay greater than that in the design with module size *R*_0_.

In the case *R*_0_ ≪ *R*, by applying Theorem 1 to any module one can see that converting that module from HD to segregated designs can reduce local conduction delay. For example, fasciculating global axons within that module into multiple tracts would reduce local conduction delay.

In the other case, if modules with size *R*_0_ contain only global axons from the neurons inside the module, by applying Theorem 1 one can see that any optimal segregated designs containing modules with size *R* ≪ *R*_0_ is equivalent to designs containing modules with size *R*_0_.

Moreover, if the tracts inside the module of size *R*_0_ contain external global axons (i.e., global axons that do not belong to the neurons inside the module with size *R*_0_ and/or do not innervate the neurons inside the module), converting segregated designs with module size *R* ≪ *R*_0_ to designs with module size *R*_0_ reduces the local conduction delay. This happens because merging all the tracts within the module of size *R*_0_ into one reduces the boundary effect. To see this, note that the minimal surface area of the big tract inside the module with size *R*_0_ is on the order of (∑Φ*_i_*)^1/2^ *R*_0_ ≪ ∑(Φ*_i_* ^1/2^)*R*_0_, where Φ*_i_* is the mean cross-sectional area of a small tract containing external global axons, and ∑(Φ*_i_* ^1/2^)*R*_0_ is the total surface area of the smaller tracts inside the module with size *R*_0_. Even if the tracts run in different directions, most of the tracts can be merged together at the scale *R*_0_, because the typical length of a tract is much greater than that, and a small curvature would not affect the total length by an order of magnitude.

Taken together, by considering the two possible cases, we have proven that the minimum conduction delay in segregated designs is achieved with module size *R*_0_. Such designs may be further classified by the relative dimensions of the gray matter. The total boundary area between gray and white matter (i.e., the total surface area of the white matter tracts), *A,* could satisfy either *A* ~ *G*/*R*_0_ or *A* ≪ *G*/*R*_0_. As the local conduction delay through the boundary effect grows with *A,* the latter design has the shorter delay. In the following, we call segregated designs satisfying *A* ≪ *G*/*R*_0_ the perforated design (PD).

### Branching Pipe Design—An Example of Perforated Design

In the previous section, we have shown that in the optimal segregated designs, the size of the module, in which global and local connections are finely intermixed, is given by *R*_0_. However, Theorem 2 does not specify other dimensions of the segregated design, such as the total surface area of the white matter tracts. In this section, by considering a specific example, which we name the branching pipe design, we show that the condition *A* ≪ *G*/*R*_0_ can be satisfied in the regime in which our perturbation approach is valid. In other words, we prove that PD exists in the regime *ND* ^2^ ≪ *G*/ℓ.

**Figure 4:**
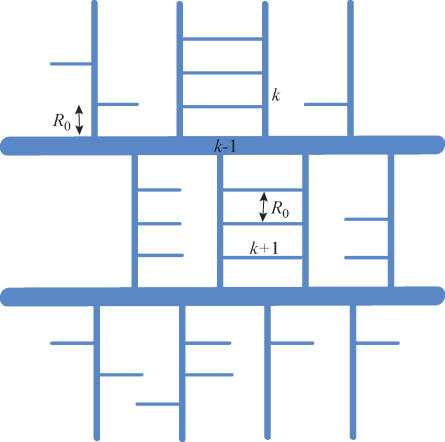
Branching Pipe Design Schematic illustration of branching pipe design with three orders of branches. The distance between *k*th order branches determines the length of the *k* + 1st-order branches. The distance between highest-order branches is given by *R*_0_.

We specify the branching pipe design as follows (Figure 4). Global axons belong to several cylindrical white matter pipes perforating the gray matter. Higher-order branches split off lower-order pipes at regular intervals. Different order branches have different lengths and different pipe diameter. The length of the zeroth-order branches (i.e., the main pipes) is given by the linear size of the brain. The length of *k* + 1st-order branches is given by the interpipe distance among the *k*th order branches, forming a space-filling structure. The interpipe distance among the finest branches is given by *R*_0_ in Equation 20 (Figure 4).

Although we can calculate the total surface area of the branching pipes for any given order *k* (as discussed in the fourth section in Materials and Methods), for simplicity we present the main results from the branching pipe design in which only first-order branches exist. We minimize the total surface area of such branching pipes and the local conduction delay by searching for the optimal length and the diameter of the first-order branches and the optimal diameter of zeroth-order branches.

We find that the expression for the minimal total surface area of the first-order branching pipes *A* depends on whether the total white matter volume is greater than the total gray matter volume or not. In the regime ℓ^2^ ≪ *ND* ^2^ ≪ *G* ^2/3^, the gray matter occupies most of the brain volume, and *A* is calculated (see the fourth section in Materials and Methods) as:


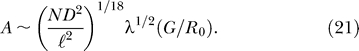


In turn, *λ* can be found by substituting *G* ~ (*N*/*n*) ℓ^3^ and optimal *R* ~ *R*_0_ (Equation 20) into Equation 16:


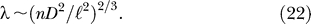


Then the minimal local conduction delay is given by


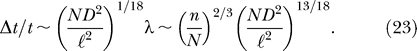


**Figure 5:**
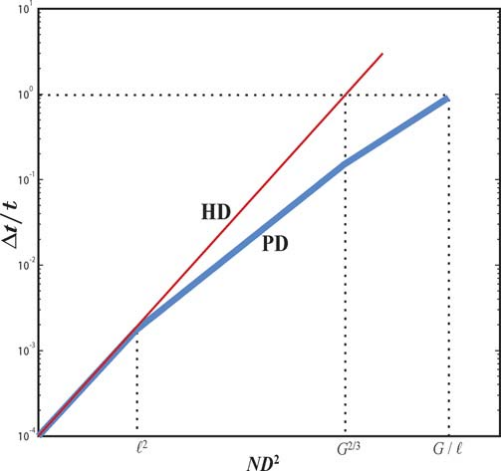
Local Conduction Delay As a Function of Global Axon Diameter in HD and PD Local conduction delay is calculated for specific values ℓ = 0.5 mm, *N* = 10^8^, and *G* = 10^3^ mm^3^ and plotted in log-log coordinates. Thin red line, local conduction delay in HD; thick blue line, local conduction delay in PD. Delay in PD is calculated for the branching pipe design containing only first-order branches.

This dependence of Δ*t*/*t* on *ND* ^2^ is plotted on log-log scale in Figure 5 (represented by the thick blue line).

In the regime *G* ^2/3^ ≪ *ND* ^2^ ≪ *G*/ℓ*,* white matter occupies most of the volume, and the specified segregated design has a different appearance: The gray matter is confined to a thin sheet. Sheet thickness is given by the length of the highest-order branches. Then, the minimum surface area of the branching pipes (as calculated in the fourth section in Materials and Methods) is given by


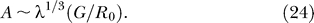


In this regime, the minimal local conduction delay is given by (Figure 5)


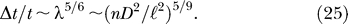


As *λ* ≪ 1 is equivalent to *ND* ^2^ ≪ *G*/ℓ (to see this, substitute *G* ~ (*N*/*n*)ℓ^3^ into *ND* ^2^ ≪ *G*/ℓ and compare it with Equation 22), we show that for such a branching pipe design, *A* ≪ *G*/*R*_0_ in the regime where our perturbation approach is valid. In other words, we verify the existence of PD in the regime *ND* ^2^ ≪ *G*/ℓ.

We note that when *λ* is approaching one, according to Equations 20 and 22, *R*_0_^2^ ~ ℓ^2^ ~ *nD* ^2^, meaning that the total surface area of the gray matter with size ℓ is taken up by the global axons. Therefore, when *λ* → 1, we must have *A* ~ *G*/*R*_0_ ~ *G*/ℓ ~ *ND* ^2^. This can also be seen from the expressions for *A* in the branching pipe design, i.e., Equations 21 and 24. Moreover, *λ* ~ 1 (i.e., *ND* ^2^ ~ *G*/ℓ) is when our perturbation approach to calculating the local conduction delay in PD breaks down (Figure 5).

When *ND* ^2^ ≫ *G*/ℓ*,* i.e., *λ* ≫ 1, we may consider clusters with discrete spatial arrangement, and each cluster has *n* neurons to implement local connectivity. In this case, we can estimate the lower limit of the cluster size, given by *n* ^1/2^ *D,* assuming that cluster volume is filled by tightly packed global axons. Because of local connections, the actual cluster size must be even greater. Alternatively, clusters may abut each other to form a sheet, and the sheet thickness could be much smaller than ℓ. In this case, however, we cannot determine the necessary conditions for the design to be optimal. Fortunately, existing anatomical data suggest that actual brains are not even close to the regime where *λ* ≫ 1, as will be shown later.

### Phase Diagram of Optimal Designs

**Figure 6:**
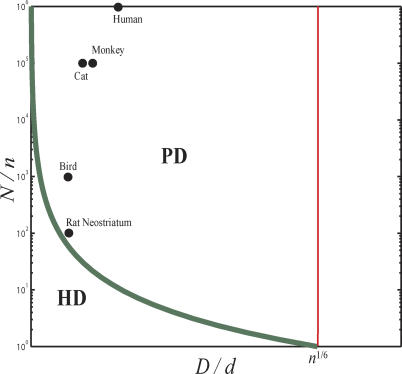
Phase Diagram of Optimal Designs In this phase diagram, we show parameter regimes in which HD or PD are optimal in terms of the global axon diameter *D,* local wire diameter *d,* total neuron number *N,* and the number of local connections per neuron *n*. We assume *n* = 10^4^ and *d* = 1 μm for all empirical data points. Values of *D* in mammalian brains are from S. S. H. Wang (personal communication) and [60], and values of *N* in the neocortex are from [44]. Value of *N* in rat neostriatum is from [62]. For birds, we assume *N* = 10^7^.

In previous sections we derived conditions under which various designs are optimal in terms of minimizing conduction delays. Specifically, HD is optimal if *ND* ^2^ ≪ ℓ^2^ and PD is optimal if *ND* ^2^ ≫ ℓ^2^ and λ ≪ 1. Next, we illustrate these results on a phase diagram (Figure 6) in terms of basic network parameters such as the local wire diameter *d,* the number of local connections (via potential synapses) per neuron *n,* global axon diameter *D,* and the total number of neurons in the brain *N*. To obtain the phase diagram, in the first-order perturbation theory, we substitute the expression for ℓ (Equations 4 and 5) into *ND* ^2^ ≫ ℓ^2^, and find that PD is optimal when (*N*/*n*)^1/2^ *D*/*n* ^1/6^ *d* ≫ 1. In the linear-log space of Figure 6, this expression corresponds to the regime above the thick green line.

Next, we estimate where perturbation theory fails by setting *λ* to one. By substituting Equations 4 and 5 into the expression for *λ* (Equation 22), we find that *λ* can be rewritten as


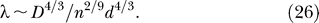


Then condition *λ* ~ 1 is equivalent to *n* ^1/6^ *d*/*D* ~ 1, corresponding to the thin red line in Figure 6.

## Discussion

We have shown that the segregation of the brain into gray and white matter may be a natural consequence of minimizing conduction delay in a highly interconnected neuronal network. We related the optimal brain design to the basic parameters of the network, such as the numbers of neurons and connections between them, as well as wire diameters. Although we do not know whether competing desiderata of short time delay and high interconnectivity were crucial factors driving evolution of vertebrate brains, our theory makes testable predictions. Below, we compare these predictions with known anatomical facts.

### Scaling Estimate of the Cortical Thickness

As fasciculated fibers are usually not observed in neocortical gray matter (according to Nissl and myelin stains), we identify cortical thickness with gray matter module size, *R*. Our prediction for the optimal module size *R*_0_ (Equation 20) can be rewritten by using Equations 4 and 5


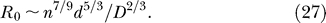


Using *n* ~ 10^4^ [22,31], *d* ~ 1 μm [31], and *D* ~ 1 μm [31] (also measured in the corpus callosum of macaque monkey; S. S.-H Wang, personal communication), we predict cortical thickness *R*_0_ ~ 1 mm. This estimate agrees well with the existing anatomical data [45,51,52], despite being derived using scaling. By substituting these values into Equation 26, we find that *λ* is smaller than one, justifying our perturbation theory approach.

Next, we apply our results to the allometric scaling relationship between cortical thickness, *R*_0_, and brain volume, *V*. We assume that *n* and *D* both increase with brain size [8,39,40] according to the following power laws: *n* ~ *V* ^1/3^ [8,39,40,44] and *D* ~ *V* ^1/6^ (see the fifth section in Materials and Methods). Then, by using Equation 27 and the constancy of the optimal local wire diameter *d* across different species [31], we predict that *R*_0_ ~ *V* ^4/27^. This prediction agrees well with the empirically obtained power law relationship (with exponent 1/9) between cortical thickness and brain volume [39,45,51–53]. Thus, our theory explains why the cortical thickness changes little while brain volume varies by several orders of magnitude between different species.

Two previous studies [39,53] also discussed the nature of the scaling law between cortical thickness and brain volume. One study [39] relies on the assumption that the number of neurons in a module of the neocortex is constant. The volume of the module might be cubic *R*_0_. Because the neuronal density may scale inversely as the cubic root of brain volume (see the fifth section in Materials and Methods), *R*_0_ should scale as one-ninth of the brain volume to ensure that the number of neurons in a module is independent of brain volume. The other study [53] relies on the assumption that the number of such modules scales as two-thirds of the total gray matter volume. Hence, the volume of the module scales as one third of the gray matter volume. As the total cortical gray matter volume may scale linearly with the brain volume (see the fifth section in Materials and Methods), the size of the module scales as one-ninth of the brain volume. In this paper, we take a different approach by deriving the expression for the cortical thickness based on the optimization principle. However, we obtain a scaling exponent close to, but not exactly equal to, one-ninth.

### Comparison of the Cortical Structure and PD

Neocortex has a sheet-like appearance, and the total area of the gray and white matter boundary is given by *A* ~ *G*/*R*_0_, where *G* is the total gray matter volume. According to our theory, such design is optimal when *λ* becomes close to one, which may be the case in big brains. Cortical convolutions may correspond to the geometry expected in the pipe design. However, when *λ* ≪ 1, our theory predicts that the optimal design satisfies *A* ≪ *G*/*R*_0_. This prediction does not seem to be consistent with empirical observations from small brains, such as the smooth, sheet-like mouse cortex. It would be interesting to know whether different requirements on connectivity or other developmental and/or functional constraints could resolve this discrepancy.

### Comparison of Mammalian Neostriatum and PD

Neostriatum is named for its striated appearance (in Nissl- and myelin-stained material [54,55]) caused by axons of neostriatal neurons gathering into fiber fascicles and perforating the gray matter [56]. Areas with higher cell density, or lower global fiber density (myelin-poor [54,55]), are called striosomes or patches [57–59]. Because this structure resembles PD, we identify patch size with *R*_0_ (Equation 27). In a typical rodent (rat or mouse) neostriatum, each principal neuron may locally contact thousands other neurons [56]. Taking *n* ~ 10^3^, *d* ~ 1 μm [31], and *D* ~ 0.6 μm [60], we estimate that *R*_0_ ~ 300 μm. This estimate agrees well with existing anatomical data [61]. In addition, we may estimate the average axonal fascicle size. Given the total number of neurons in the rat neostriatum is about 10^6^ [62], we find that the fascicle diameter is of the same order of ℓ, approximately 100 μm (see Equation 58 in the fourth section in Materials and Methods). This estimate agrees well with fascicle size [55] (see also http://www.hms.harvard.edu/research/brain/atlas.html).

### Comparison of the Avian Telencephalon and PD

Bird brains also exhibit segregation into gray and white matter and may resemble PD. Distinct fiber fascicles have been identified that connect different brain regions (see http://avianbrain.org/boundaries.html), such as the connections from HVC to RA in songbirds, which are presumably myelinated axons [63]. Interestingly, unlike in mammals, which have a large cortex on the top of other brain structures, in birds the white matter fascicles can be scattered throughout the whole forebrain. However, more precise data would be desirable, such as measurements of large-scale myelin distribution in serial sections of bird telencephalons.

### Comparison of the Spinal Cord and PD

While the inner core of the spinal cord contains gray matter, the outer shell contains the white matter consisting of long axons from spinal and cortical neurons [56]. According to our theory, such organization is optimal if the inner core diameter is on the same order as *R*_0_. To see if this is the case, note that a principal (motor) neuron in the spinal cord has a very large arbor span [56,64] and may receive 10^5^–10^6^ potential connections. Given *n* ~ 10^5^, *d* ~ 1 μm, and *D* ~ 1 μm, we find *R*_0_ ~ 8 mm according to Equation 27, which is on the same order as the inner core diameter [56].

### Related Work

Our work builds upon several insights from recent studies. In particular, the idea of minimizing conduction delay has been used to explain why axons and dendrites take a certain fraction of the neuropil [17]. The main result in that paper is further extended in this study to show that local conduction delay must increase after mixing gray and white matter (see Materials and Methods). Also, in our model local circuits are approximated by the network with all-to-all connectivity, which relies on the concept of potential synapses [23]. Adopting this model allowed us to derive explicit results for the total length of local connections (see the first section in Materials and Methods) [6].

We benefited from several previous studies of anatomical and functional connectivity between different cortical areas. These studies helped conceptualize network connectivity by revealing many interesting features of the network [65–71], such as hierarchal [72], clustering [73], and small-world properties [41,74], which helped to generate new models to address functional specialization and integration [75–80].

We adopted (with the potential synapse caveat) the connectivity model used by Ruppin et al. [19] and Murre and Sturdy [20]. These authors applied the wiring optimization approach to explain the segregation of white and gray matter in the brain. Given a network with local and global connections, they searched for a design having minimum total wiring volume. They attempted to show that a segregated cortex-like design has a smaller volume than does a homogeneous structure.

Murre and Sturdy [20] used the scaling approach to calculate network volume for several network connectivity patterns and layouts. We verified their calculation of the interior (homogeneous) structure volume. However, their calculation of the external (cortex-like) structure volume does not seem to be self-consistent. The volume of axons in the external structure was calculated by using the expression that was unjustifiably adapted from the internal structure calculation, thus undermining their conclusion.

Ruppin et al. [19] did not rely on scaling arguments and calculated the volume of brain structures given their geometric characteristics, under reasonable assumptions of connectivity parameters. These authors showed that segregation of the network into the inner core organization, which has an inner core of gray matter surrounded by white matter, does not lead to volume efficiency compared to a homogeneous structure. They also showed that the external sheet (cortex-like) structure has a smaller volume than the inner core organization. However, this does not prove that the cortex-like structure has a smaller volume than the homogeneous structure, a conclusion relying on a fine balance of numerical factors.

We analyzed the advantages of gray and white matter segregation from the conduction delay perspective. Our results complement previous studies in some respects but differ in many others. Here, we summarize several novel points. First, we showed that the segregation of white and gray matter is consistent with minimizing conduction delay. Second, we determined the maximum number of neurons in the all-to-all connected network with a reasonable conduction delay and showed that local cortical networks are close to that limit. Third, we proposed a possible explanation for the thickness of the neocortex, which varies surprisingly little among mammalian species. Unlike Murre and Sturdy [20], who suggested that cortical thickness is determined by the maximum density of incoming and outgoing global axons (condition indicated by the thin red line in Figure 6), we argue that in most brains it is the result of minimizing local conduction delay. Fourth, our theory is based on the scaling approach and yields a phase diagram of optimal designs for a wide range of parameters. This allowed us to apply the theory to several different structures other than the neocortex. Derived scaling relationships can be tested by future experimental measurements.

### Wiring Volume and Conduction Delay Minimization

As features of brain design have been explained by minimizing both the total volume and the conduction time delay, it is natural to wonder how these approaches relate to each other. In general, the evolutionary cost is likely to include both the volume and the time delay. Hopefully, such unified framework will emerge eventually. In the meanwhile, since the exact form of the cost function is not known, we sought to construct theories to explain features of brain architecture based on the simplest possible assumptions. Next, we proposed how time delay and volume can be related based on the current theory.

In our model, conduction delay in local circuits is minimal when the local wire diameter is at its optimal value, which corresponds to an optimum gray matter volume. (For details, see the first section in Results.) The local conduction delay increases when the local wire diameter *d* is smaller than the optimum value. In this case, volume cost and conduction delay cost are competing requirements. In the opposite case, when the local wire diameter is thicker than the optimal value, invoking additional conduction delay cost is accompanied by additional volume cost. Therefore, as long as the gray matter volume is greater than its optimal volume, e.g., because of intermixing global axons with gray matter, we may associate the additional conduction delay cost with the volume cost, named the effective volume cost.

However, in the white matter, the relationship between volume and delay is different. Increasing white matter volume by making the global axon diameter thicker does not increase the global conduction delay (see the second section in Materials and Methods). Thus, the effective volume cost of white matter is just the tissue cost. From this perspective, we propose that gray matter has a greater effective volume cost than does white matter. This may have several biological implications: (1) Initial segments of axons originating from pyramidal neurons head straight toward (and are perpendicular to) the boundary between the white and gray matter. Once axons cross the white/gray matter border, they change direction. Although such design may increase the length of global axons, it largely reduces the effective volume cost of gray matter, because the volume of global axons in the gray matter is minimal. (2) Another implication of differential effective volume costs in the gray and white matter is that the global axons in gray matter may be thinner than in white matter. Such variation in diameter could preserve short conduction delays in local and global connection. Of course, global axons cannot be made infinitesimally small without sacrificing global conduction delay. Further exploration of this effect would require more experimental measurements of diameter changes at the white/gray matter border. (3) In abutting topographically organized cortical sensory areas, the maps are mirror reflections of each other relative to the border of the areas. The purpose of such organization remains unclear, because interarea connections in the white matter do not benefit from this organization. In particular, placing two cortical areas next to each other (without mirror reflection) would not increase the length of interarea connections in the white matter. Yet, according to our theory, neurons close to the border would be at a disadvantage, because their local connections would have to reach further to find appropriate targets. Mirror-reflecting maps relative to the interarea border would eliminate a discontinuity in a map and place neurons with similar receptive fields closer to each other. Such an arrangement would benefit intracortical connections.

## Materials and Methods

### Minimization of conduction delay in a local network with branching axon and dendrite design.

Here we revisit the analysis from [17] using more specific information about the network. Consider wiring up a local network of *n* neurons with all-to-all potential connectivity. The mean conduction delay in local circuits is given by


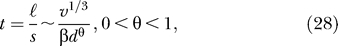


where *d* is the local wire diameter; *v* ^1/3^, the linear size of the local network, approximates the average path length between two potentially connect two neurons. We assume a sublinear relationship between local wire diameter and conduction velocity, and *β* is a proportionality coefficient. From Equation 28, we want to find the minimal local conduction delay and the corresponding optimal local network volume. Therefore, we have to eliminate wire diameter *d* from the previous equation and rewrite it as a function of local network volume. To get this expression, we first notice that the total volume of the local network is given by


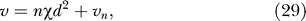


where *v_n_* is the nonwire volume, which is assumed to be a constant, and *χ* is the total wire length per neuron. Second, for an all-to-all potentially connected network, by applying the branching axon and dendrite design [6], we also have


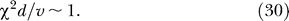


This expression is derived as follows [6]. First, the local network volume, *v,* is divided into cubes of volume, *d* ^3^, i.e., into *v*/*d* ^3^ voxels. Then, the number of potential contacts between an axon and a dendrite is given by the number of voxels that contain them both. Each axon occupies *χ*/*d* voxels, the same number as a dendrite. The fraction of voxels containing the axon is (*χ*/*d*)/(*v*/*d* ^3^), the same as the fraction containing the dendrite. Then, the fraction of voxels containing both the axon and the dendrite is the product of the two fractions, *χ* ^2^ *d* ^4^/*v* ^2^. By multiplying this fraction by the total number of voxels, we find the number of voxels containing axon and dendrite, *χ* ^2^ *d*/*v*. Then, the condition for having at least one potential contact is given by Equation 30. Combining Equation 29 with Equation 30 and excluding *χ* yields


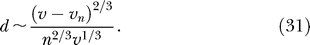


By combining Equation 28 with Equation 31, we obtain


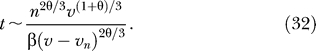


In Equation 32, by setting the first derivative of *v* to zero, we find the optimal network volume, or gray matter volume, should be


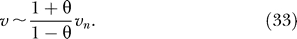


And the minimal local conduction delay is given by


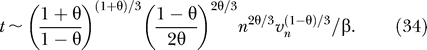


We assume that nonwire consists mostly of synaptic components, such as axonal boutons and spine heads. In addition, only a fraction, *f*(0.1–0.3), of potential synapses are actual synapses [23]. Therefore, the nonwire volume can be estimated as


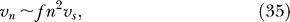


where *v_s_* is a single synapse volume. Assuming that *θ* = 1/2 from classical cable theory and substituting it into Equations 34 and 35, we find the minimal local conduction delay is proportional to


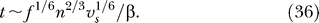


For simplicity, after neglecting *f,* this expression is used in Equation 6. Furthermore, the optimal wire diameter can also be calculated by combining Equations 31, 33, and 35, which gives


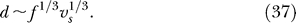


After neglecting *f,* this expression also appears in Equation 5.

### Global conduction delay can be preserved after intermixing gray and white matter.

After introducing the local connections (gray matter) into the global connections, the total network volume swells and Equation 11 changes to


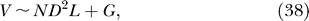


where *G* is the total gray matter volume. After substituting *L* ~ *V* ^1/3^, *D* ~ *L*/(*BT*), i.e., Equation 9 and 10, into Equation 38, the expression for *V* can be rewritten as


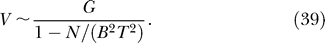


After substituting Equation 39 into *D* ~ *L*/(*BT*) ~ *V* ^1/3^/(*BT*), we find the global axon diameter is given by


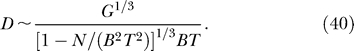


Therefore, as long as *T* > *N* ^1/2^/*B*, we can find the corresponding global axon diameter *D*.

### Local conduction delay increases after intermixing gray and white matter.

Consider again the network described in above with *n* neurons and all-to-all potential connectivity. After white matter perforates the neuropil, its volume inside gray matter can be expressed by *vλ,* where *v* is the unperturbed optimal local gray matter volume given by Equation 33 and *λ* is a positive dimensionless parameter. After such perturbation, the volume of the local network, i.e., Equation 29, changes to


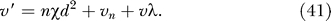


Second, for an all-to-all potentially connected network, by applying the branching axon and dendrite design [6], Equation 30 changes to


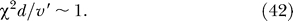


By combining Equations 28, 41, and 42 and excluding *χ* and *d,* we can express the local conduction delay as a function of the total local network volume *v*′:


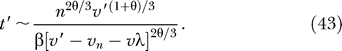


Equation 43 shows that *t*′ is a monotonically increasing function of *λ,* and we recover the expression for *t* in Equation 32 as *λ* = 0. Moreover, when *λ* ≪ 1, the local network is still close to the unperturbed optimal state, i.e., *v*′ ≃ *v*, and we can expand Equation 43 to the first order of *λ,* which yields


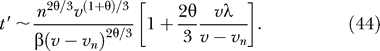


After combining Equation 44 with Equations 32 and 33, we obtain the expression for local conduction delay from the perturbation theory,


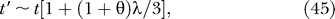


or


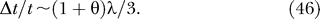


After neglecting the numerical coefficient in the spirit of scaling estimate, the last expression also appears in Equation 14.

### Local conduction delay and surface area in the branching pipe design.

We will address stepwise the process by which we developed this design; first, we present general considerations; second, we develop the first-order branching design; and third, we describe the nonbranching pipes design.

First, to calculate the local conduction delay in the branching pipes, we consider a general model in which the white matter pipes have total *J* branching orders. A branch at order *k* (0 ≤ *k* ≤ *J*) has length *L_k_* and pipe diameter *P_k_*. The total number of *k*th order branches within the neuropil with linear size *L_k_* is given by *M_k_*. Then, we can evaluate the relative local conduction delay increase through the boundary effect introduced by the *k*th order branches. The affected neuropil volume through the boundary effect is given by the product of total pipe surface area, *M_k_P_k_L_k_,* and distance ℓ. This means that the ratio of the affected volume to the total gray matter volume, or the relative local conduction delay increase is given by


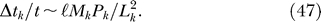


However, Equation 47 does not tell us what the total local conduction delay is, as different branching orders can have different branching length and diameter.

To examine this further, we assume that the branching structure has a space-filling feature. In particular, the length of the main branch *L*_0_ is given by the linear size of the network, *G* ^1/3^, and the length of *k* + 1st order branch is given by the interpipe distance among the *k*th order branches. For the terminal branches *k* = *J,* the interpipe distance between them is given by *R*_0_ (Equation 20).

If the length of the *k* + 1st order branches is much larger than the diameter of the *k*th order branches, i.e., *L_k_*_ + 1_ ≫ *P_k_*, the interpipe distance between *k*th order branches is given by *L_k_*/*M_k_* ^1/2^. Thus, we have


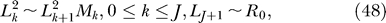


where *L_J_*_ + 1_ is the interpipe distance among the terminal branches, given by *R*_0_ (Equation 20). By denoting *N_k_* as the number of neurons in the neuropil with linear size *L_k_, N_k_* and *N_k_*_ + 1_ also have the following relationship


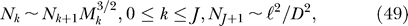


according to Equation 48, where *N_J_*_ + 1_ is the total neuron number in the neuropil with linear size *R*_0_. In addition, because the pipes with length *L_k_* contains the global axons from the neurons inside the neuropil with linear size *L_k_*, we should also have


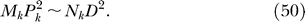


By substituting Equations 48–50 into Equation 47, we find that


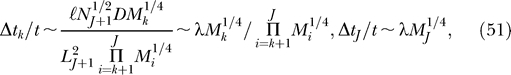


where ℓ*N_J_*_ + 1_^1/2^ *D*/*L_J_*_ + 1_^2^ ~ ℓ^2^/*R*_0_^2^ ~ *λ,* because according to Theorem 2, ℓ^2^ is the total cross-sectional area of the global axons inside the module with size *R*_0_. Then, the total local conduction delay increase through the boundary effect is given by


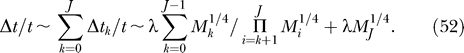


This expression can be minimized as a function of *M_k_*. As a result, we obtain


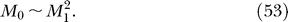


For *J* > 1, we also have


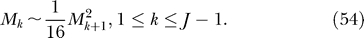


Given the total number of neurons in the gray matter *N* = *N*_0_ and the total branching orders *J,* by substituting Equations 53 and 54 into Equation 49, we can also obtain *M_k_* explicitly. Next, by using Equations 48–50, we can find the optimal branching length and diameter for different branching orders.

Second, we consider a simple branching model in which only the first-order branches exist. In this case, *J* = 1, and by substituting Equation 53 into Equation 49, we obtain


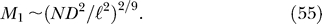


By substituting Equations 55 and 53 into Equation 52, the relative local conduction delay increase through the boundary effect is given by


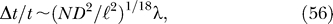


where we neglect the numerical factor of the order of one in the spirit of the scaling estimate. The total local conduction delay increase is the sum of Equation 56 and the expression for relative local conduction delay increase due to intermixing nonfasciculated global axonal segments and gray matter, i.e., *λ*. However, for the scaling estimate, the second term could be ignored, and we obtain Equation 23.

Next, we calculate the total surface area of the branching pipes *A*. According to Equation 56 and Δ*t*/*t* ~ ℓ*A*/*G*, we then obtain the total surface area of the branching pipes


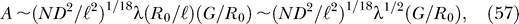


where the last expression uses the relationship ℓ^2^/*R*_0_^2^ ~ *λ*. This expression also appears in Equation 21.

In addition, we can also estimate the diameter and length of the first-order branching pipes. *P*_1_ can be obtained by combining Equations 49 and 50, which yields


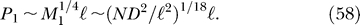


And according to Equation 48, *L*_1_ is given by


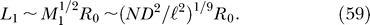


In the previous analysis, we assume that the length of the first-order branches is much larger than the diameter of the main branches, i.e., *L*_1_ ≫ *P*_0_, which allows us to use Equation 48. This assumption holds when the total white matter volume is much smaller than the gray matter volume, i.e., *ND* ^2^ ≪ *G* ^2/3^.

In the opposite regime, however, *L*_1_ ≪ *P*_0_ must hold, as the volume of the main branching pipe is much larger than the gray matter volume surrounding it. To see this, note that the volume of the main branching pipe is given by *P*_0_^2^ *L*_0_, where *L*_0_ is the length of the main branch and the volume of the gray matter surrounding an individual pipe is given by (*P*_0_ + *L*_1_)^2^ *L*_0_ − *P*_0_^2^ *L*_0_. Then, it is easy to check that if the gray matter volume is much larger than the white matter pipe volume, we have *L*_1_ ≫ *P*_0_, while in the opposite case we have *L*_1_ ≪ *P*_0_. Geometrically, when *ND* ^2^ ≫ *G* ^2/3^, the gray matter resembles a sheet, and the sheet thickness is given by the length of the first-order branches.

As the pipe design exhibits a different configuration when *ND* ^2^ ≫ *G* ^2/3^, we expect that the expressions for the total surface area of the pipes and the minimal local conduction delay are different from what we derived above. In this case, the total surface area of the main branching pipes is equal to the surface area of the gray matter sheet *G*/*L*_1_, and the relative local conduction delay increase through the boundary effect of the main branches is given by Δ*t*_0_/*t* ~ ℓ*G*/*L*_1_ *G* ~ ℓ/*L*_1_.

To calculate the boundary effect induced by the terminal branches, we assume that *R*_0_ ≫ *P*_1_, where *P*_1_ is the diameter of the terminal branches. This condition allows us to use Equations 48–50. Later, we will confirm that *R*_0_ ≫ *P*_1_ holds. Then, *L*_1_ ~ *M*_1_^1/2^ *R*_0_, *P*_1_ ~ *M*_1_^1/4^ℓ, and the relative delay increase due to the terminal branches is given by Δ*t*_1_/*t* ~ ℓ*P*_1_ *M*_1_/*L*_1_^2^ ~ *λM*_1_^1/4^. By adding up the local delay from the main and the first-order branches, we find that in the regime *ND* ^2^ ≫ *G* ^2/3^, the total local conduction delay increase is given by


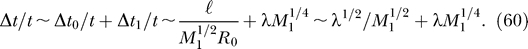


Minimizing this expression as a function of *M*_1_, we obtain *M*_1_ ~ *λ* ^−2/3^, and Δ*t*/*t* ~ *λ* ^5/6^, as appeared in Equation 25.

Next, we calculate the total surface area of the pipes *A*. As Δ*t*/*t* ~ ℓ*A*/*G* ~ *λ* ^5/6^, we then obtain the total surface area of the branching pipes


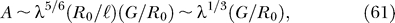


as appeared in Equation 24.

To check whether *R*_0_ ≫ *P*_1_, we note that *P*_1_ ~ *M*_1_^1/4^ℓ. Then, *R*_0_ ≫ *P*_1_ requires *R*_0_ ≫ *λ* ^−1/6^ℓ, as *M*_1_ ~ *λ* ^−2/3^. In turn, this requires *λ* ≪ 1 as ℓ/*R*_0_ ~ *λ* ^1/2^. Thus, *R*_0_ ≫ *P*_1_ if *λ* ≪ 1. This condition should always be satisfied for the PD.

Third, the nonbranching pipe model corresponds to *J* = 0. It does not belong to the PD, because *A* ≪ *G*/*R*_0_ does not always hold in such a design when *λ* ≪ 1, i.e., *ND* ^2^ ≪ *G*/ℓ. To see this, we note that in the regime *ND* ^2^ ≫ *G* ^2/3^, *A* ~ *G*/*R*_0_ must hold in the nonbranching pipe model, because the pipe diameter *P*_0_ is much larger than *R*_0_. In other words, when *G*/ℓ ≫ *ND* ^2^ ≫ *G* ^2/3^, the gray matter in the nonbranching pipe model resembles a sheet with thickness *R*_0_.

### Scaling of the mammalian neocortex.

The theoretical framework developed in this paper allows us to derive several scaling laws for the neocortex. Provided our perturbation theory is valid, the total neocortical volume *G* should be proportional to the total nonwire volume. Assuming that nonwire contains mostly synapses, we have


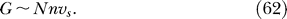


First, from Equation 62, we find that the synaptic density, *ρ_s_,* is a constant, since *ρ_s_* ~ *Nn*/*G* ~ 1/*v_s_,* where the average synapse volume *v_s_* is assumed to be a constant in different cortical areas and across different species. The prediction of constant synapse density is supported by experimental observations [31,40,81,82] from a small number of taxa so far, and was used as a starting point to derive scaling laws of the mammalian brains in several theoretical papers [39,45].

Second, we find the neuronal density *ρ* ~ *N*/*G* ~ *N*/(*Nnv_s_*) ~ 1/*n*. Since *ρ* scales inversely as the cubic root of total brain volume *V* across different mammalian species (*ρ* ~ *V* ^−1/3^) [40,83], and the cortical volume is loosely proportional to the brain volume (*G* ~ *V*) [84], we find *n* ~ *V* ^1/3^, *N* ~ *V* ^2/3^, and *n* ~ *N* ^1/2^. We note that Braintenberg [31,44] has previously proposed the square-root relationship between *n* and *N*. He assumed that the cerebral cortex could be divided into *N* ^1/2^ compartments and each compartment contains *N* ^1/2^ neurons. The local connectivity within a compartment is almost all-to-all, and every compartment is connected to every other one by a global axon.

Third, we find that the global axon diameter *D* scales as *V* ^1/6^. To see this, we note that the total white matter volume *W* is given by *ND* ^2^ *V* ^1/3^, where the average length of global axons in the white matter is assumed to be proportional to the brain size, *V* ^1/3^. Since *N* ~ *V* ^2/3^, and it has also been reported that *W* ~ *V* ^4/3^ across different mammalian species [3,39,84–86], we find *D* ~ *V* ^1/6^. This is consistent with recent measurements from corpus callosum, which indicates that the average diameter of global axons scales monotonically with the brain size [40]. Then, using *n* ~ *V* ^1/3^, *D* ~ *V* ^1/6^ and Equation 27, we obtain *R_0_* ~ *V* ^4/27^, an expression from the first section in Discussion.

## Abbreviations

HD: homogeneous design
PD: perforated design
